# Exposure Classification and Temporal Variability in Urinary Bisphenol A Concentrations among Couples in Utah—The HOPE Study

**DOI:** 10.1289/ehp.1509752

**Published:** 2015-09-15

**Authors:** Kyley J. Cox, Christina A. Porucznik, David J. Anderson, Eric M. Brozek, Kathryn M. Szczotka, Nicole M. Bailey, Diana G. Wilkins, Joseph B. Stanford

**Affiliations:** 1 Office of Cooperative Reproductive Health, Division of Public Health,; 2 Center for Human Toxicology, and; 3 Department of Pathology, University of Utah, Salt Lake City, Utah, USA

## Abstract

**Background:**

Bisphenol A (BPA) is an endocrine disruptor and potential reproductive toxicant, but results of epidemiologic studies have been mixed and have been criticized for inadequate exposure assessment that often relies on a single measurement.

**Objective:**

Our goal was to describe the distribution of BPA concentrations in serial urinary specimens, assess temporal variability, and provide estimates of exposure classification when randomly selected samples are used to predict average exposure.

**Methods:**

We collected and analyzed 2,614 urine specimens from 83 Utah couples beginning in 2012. Female participants collected daily first-morning urine specimens during one to two menstrual cycles and male partners collected specimens during the woman’s fertile window for each cycle. We measured urinary BPA concentrations and calculated geometric means (GM) for each cycle, characterized the distribution of observed values and temporal variability using intraclass correlation coefficients, and performed surrogate category analyses to determine how well repeat samples could classify exposure.

**Results:**

The GM urine BPA concentration was 2.78 ng/mL among males and 2.44 ng/mL among females. BPA had a high degree of variability among both males (ICC = 0.18; 95% CI: 0.11, 0.26) and females (ICC = 0.11; 95% CI: 0.08, 0.16). Based on our more stringent surrogate category analysis, to reach proportions ≥ 0.80 for sensitivity, specificity, and positive predictive value (PPV) among females, 6 and 10 repeat samples for the high and low tertiles, respectively, were required. For the medium tertile, specificity reached 0.87 with 10 repeat samples, but even with 11 samples, sensitivity and PPV did not exceed 0.36. Five repeat samples, among males, yielded sensitivity and PPV values ≥ 0.75 for the high and low tertiles, but, similar to females, classification for the medium tertile was less accurate.

**Conclusion:**

Repeated urinary specimens are required to characterize typical BPA exposure.

**Citation:**

Cox KJ, Porucznik CA, Anderson DJ, Brozek EM, Szczotka KM, Bailey NM, Wilkins DG, Stanford JB. 2016. Exposure classification and temporal variability in urinary bisphenol A concentrations among couples in Utah—the HOPE study. Environ Health Perspect 124:498–506; http://dx.doi.org/10.1289/ehp.1509752

## Introduction

Bisphenol A (BPA) is an endocrine-disrupting chemical with nearly ubiquitous, involuntary exposure, and is commonly found in polycarbonate plastic, epoxy resins, food can linings, dental sealants, thermal receipt paper, and other commercial products (Calafat et al. 2005; Kang et al. 2006; Lakind and Naiman 2011).

BPA has received considerable attention as a potential reproductive toxicant, but results of epidemiologic studies have been mixed and criticized for the predominance of cross-sectional study designs and inadequate exposure assessment (Lakind et al. 2014). BPA is short-lived in the body and is excreted through the urine with a half-life of ~ 6 hr (Völkel et al. 2002). Urinary concentrations reflect exposure that occurred during a relatively short period preceding sample collection, making a spot sample reflective of long-term exposure only if daily exposures are fairly constant over time. Previous studies have examined temporal variability of urinary BPA concentrations and found significant within- and between-person variability with low to moderate reproducibility over time (Arakawa et al. 2004; Braun et al. 2011, 2012; Christensen et al. 2012; Mahalingaiah et al. 2008; Meeker et al. 2013; Nepomnaschy et al. 2009; Teitelbaum et al. 2008), as well as significant variation within a day (Ye et al. 2011). Although spot samples are resource efficient, they are likely to introduce misclassification bias via the inability to measure transient exposures and exposures that are rapidly excreted. Studies of BPA exposure and health outcomes have been limited by reliance on a single exposure measurement as a proxy for an entire developmental stage, such as preconception, the trimesters of pregnancy, or the postpartum period (Braun et al. 2014; Lee et al. 2014; Perera et al. 2012; Valvi et al. 2013).

The aim of this study was to assess exposure classification and temporal variability among individuals with repeated first-morning urinary BPA measurements collected over one to two menstrual cycles.

## Methods

*Participants.* This analysis was performed on a subset of participants (83 couples, 166 participants) from an ongoing prospective cohort of heterosexual couples planning pregnancy within 3 months after enrollment—the Home Observation of Periconceptional Exposures (HOPE) Study. Participants were recruited from the greater Salt Lake City, Utah, area beginning in January 2012. Female participants were required to be 18–35 years of age and male participants 18–40 years of age.

The study was approved by the University of Utah Institutional Review Board, and participants signed an informed consent document before participation. Following consent, participants met with a member of the study staff who explained study procedures, provided biospecimen collection materials, and obtained height and weight measurements.

*Urine sample collection.* Following our protocol using a previously validated method for identifying ovulation, female participants observed changes in cervical mucus and identified an estimated day of ovulation (EDO) and fertile window (time when conception is likely to occur) for each menstrual cycle (Porucznik et al. 2014). Male and female participants collected daily first-morning urine samples (first void upon waking) from the first day of fertile-quality cervical mucus. Males discontinued collecting after the EDO, making their total collection window approximately 7 days and corresponding to their female partner’s fertile window. Females continued to collect for the remainder of the menstrual cycle up until the onset of the next menses or until she had a positive pregnancy test, which was performed 18 days following the EDO. In cases where first-morning samples were not collected, participants were instructed to collect later in the day and mark the specimen to indicate that it was not a first-morning void. Using the same collection protocol, both partners collected urine samples for a second cycle if pregnancy was not achieved during the first cycle. Urine was collected in 4-oz polypropylene specimen cups and then transferred to 50-mL polypropylene tubes that were placed in the participants’ home freezer until the end of the menstrual cycle. At the end of each cycle, a member of the study staff collected the samples from the participants and transported them to the Center for Human Toxicology at the University of Utah for analysis.

*Urinary BPA measurements.* Urine samples were stored at –20°C upon arrival at the lab and again after processing. Total BPA (unconjugated BPA plus mono-glucuronide conjugate and mono-sulfate conjugate) was measured in the urine samples using ultra-high-performance liquid chromatography–tandem mass spectrometry. Analytical chemistry methods and quality control procedures have previously been described (Anderson et al. 2014). Briefly, the method used liquid/liquid extraction with 1-chlorobutane and a human urine aliquot sample of 800 μL. Chromatography was performed on an Acquity UPLC® system (Waters Corporation, Milford, MA) with a Kinetex® Phenyl-Hexyl column (Phenomenex, Torrance, CA). Mass spectrometric analysis was performed with negative electrospray ionization on a Quattro Premier XE^TM^ (Waters Corporation). Acceptance criteria for analytical standards and quality controls were ± 20% of nominal concentration. The limit of detection (LOD) was 0.1 ng/mL and the limit of quantification (LOQ) was 0.75 ng/mL. All samples from members of the same couple (per cycle) were analyzed concurrently during the same analytical batch. To prevent contamination, laboratory glassware, consumables, and reagent chemicals were verified not to be sources of BPA via laboratory assay before use. Biospecimen collection materials were made of polypropylene, according to manufacturer specifications, which is not expected to be a source of BPA.

*Statistical analysis.* Analysis was performed using SAS software (version 9.3; SAS Institute Inc., Cary, NC). A total of 296 (11.3%) values were below the LOQ and were assigned a value of LOQ divided by the square root of 2, or 0.53 ng/mL (Hornung and Reed 1990). Because the number of urine samples from each participant varied, and within-person concentrations were log-normally distributed, geometric means (GM) with 95% confidence intervals (CI) are presented.

These data are uncorrected for urinary dilution. The type of correction used to adjust for urinary dilution in BPA literature is inconsistent. It is common to see adjustment for specific gravity, urinary creatinine or osmolality. However, Lassen et al. (2013) report that no methods used to adjust for urinary dilution (osmolality, volume, creatinine) altered the consistency of repeated measures among comparison of two spot, three first-morning, and three 24-hr urine samples collected over a 3-month time period, although these samples were collected from 33 Danish males and their study did not include females. A sensitivity analysis performed on a subset of HOPE specimens (453 samples from females, 42 samples from males) found that correction for urinary dilution of predominantly first-morning samples, using both specific gravity and urinary creatinine, did not appreciably alter the concentrations (mean 19% relative change) and did not change relative tertile categorization into tertiles of exposure for each participant (data not shown). Given that tertile categorization in surrogate category analysis and analysis of repeated measures is the primary focus of this paper and we have repeated first-morning samples, we elected not to correct for urinary dilution in these analyses.

Given that BPA exposure occurs primarily via ingestion of food, observations from members of the same couple who share a common household diet cannot be considered independent (Mahalingaiah et al. 2008). Additionally, multiple observations from the same individual cannot be considered independent. To account for these factors, clustering at the individual and male–female partner levels was used when calculating geometric means for the cohort using SAS PROC SURVEYMEANS.

*Intraclass correlation coefficient analysis.* To quantitatively assess between- and within-subject variability of urinary BPA concentrations, intraclass correlation coefficients (ICCs) and their 95% CIs were calculated using mixed random effects models that accounted for clustering at the male–female partner level and for multiple cycles, when appropriate, in SAS PROC MIXED (Hankinson et al. 1995; Rosner 2010). ICC is a measure of the reliability of repeated measures over time, defined as the ratio of the between-subject variance and total variance (sum of between-subject variance and within-subject variance). ICC ranges from zero to one, with values near zero indicating poor reliability and a greater degree of variation within subjects, and values near one indicating high reliability and a greater degree of variation between subjects (Rosner 2010).

*Exposure classification analysis.* Although the ICC is an indicator of reliability for continuous values, it does not measure the extent of exposure misclassification that may occur if exposure is categorized—for example, into tertiles of low, medium, and high exposure. To address this and examine how the number of samples collected may improve categorization, we performed surrogate category analyses (Braun et al. 2012; Hauser et al. 2004; Mahalingaiah et al. 2008; Meeker et al. 2012). We began by calculating the GM across all the samples collected for each participant separating each cycle/month, where applicable, and considered this the “true” or most accurate assessment of the individual’s mean exposure. We then classified the “true” GM into a low, medium, or high tertile of exposure. Among participants who collected samples for more than one cycle, a separate GM and tertile was calculated for each cycle. Tertiles were created separately for males and females based on the sex-specific distribution of BPA geometric means of all samples, and males and females were analyzed separately in this analysis. Participants were then classified into “predicted” or surrogate tertiles based on the concentration of a subset of randomly selected samples from their corresponding cycle-specific pool of total samples.

To assess how adding additional samples improved categorization, we sequentially added samples to the surrogate GM and then categorized that into a surrogate tertile. The procedure was repeated, adding one more specimen each time until *n* – 1 was reached for the available specimens for that individual and cycle—performing this procedure separately for each cycle that the participant collected specimens. GMs of all possible combinations of sample pairings in each of these levels and for each participant/cycle were used when calculating the “surrogate” value. The surrogate tertile was then compared to the “true” tertile in a contingency table. Values from individual contingency tables were combined into a single table, and overall sensitivity, specificity, and positive predictive value (PPV) were calculated and weighted by the effective sample size (the number of possible combinations per person multiplied by the number of individuals in each stratum). The goal of this analysis was to simulate and compare the ability of exposure assessment that involves a single sample, or set of repeated samples, to predict a female’s “true” exposure over a single menstrual cycle and a male’s exposure over a single fertile window. This version of the surrogate category analysis, hereafter referred to as analysis 1, is similar to previous studies (Braun et al. 2012; Hauser et al. 2004; Mahalingaiah et al. 2008).

The surrogate category analysis was repeated with slightly different methods. In the second version, hereafter referred to as analysis 2, the samples used to calculate the surrogate GM were not used in the calculation of the “true” GM. This eliminates the structural dependency that inherently exists between the surrogate GM and the “true” GM when the samples used in the surrogate GM are also included in the “true” GM (Braun et al. 2012; Mahalingaiah et al. 2008). However, once more than half the sample values are used in the calculation of the surrogate GM (and the remaining values are used to calculate the “true” GM), the values obtained for sensitivity, specificity, and PPV are reciprocal complements to the values already obtained, and thus, were not included in the final calculations. For example, if a person had a total of 20 samples and 1 was used to calculate the surrogate GM, the remaining 19 would be used to calculate the “true” GM. As incrementing to *n* – 1 continued, eventually the surrogate GM would include 19 specimens leaving only 1 to be used in the calculation of the “true” GM. This calculation would produce reciprocal values complementary to those obtained in the previous 19 versus 1 calculation. Therefore, surrogate values for this approach were only done for up to *n*/2 samples for any individual. In instances where no surrogate values fell into a particular “true” tertile, sensitivity and PPV could not be calculated for that tertile, so the total number of observations contributing to each final mean differed. We used this method to avoid the problem of overinflation of the sensitivity, specificity, and PPV that occurs due to the non-independence of the surrogate and “true” measures with analysis 1 (Braun et al. 2012; Mahalingaiah et al. 2008).

## Results

The mean age of female participants was 26.8 ± 3.8 years and the mean for male participants was 28.3 ± 4.0 years (Table 1). Most participants (144, 86.7%) were Caucasian and only 6 (3.6%) were Hispanic, with mean body mass index (BMI) of 25.3 ± 5.3 kg/m^2^. This population was highly educated, with 60.8% (*n =* 101) college graduates and 36.1% (*n =* 60) with some college education (1–3 years). Most participants were never smokers (149, 89.8%).

**Table 1:** Characteristics of study participants [mean ± SD or *n* (%)].

Characteristic	All subjects (*n *= 166)	Male (*n *= 83)	Female (*n *= 83)
Age (years)	27.5 ± 4.0	28.3 ± 4.0	26.8 ± 3.8
< 25	42 (25.3)	15 (18.1)	27 (32.5)
25–35	120 (72.3)	64 (77.1)	56 (67.5)
> 35	4 (2.4)	4 (4.8)	0
BMI^*a*^	25.3 ± 5.3	26.1 ± 4.7	24.4 ± 4.9
< 18.5	4 (2.4)	0	4 (4.8)
18.5–24.9	88 (53.0)	37 (44.6)	51 (61.5)
25.0–29.9	45 (27.1)	30 (36.1)	15 (18.1)
≥ 30	28 (16.9)	15 (18.1)	13 (15.7)
Missing	1 (0.6)	1 (1.2)	0
Race
Caucasian	144 (86.7)	71 (85.5)	73 (88.0)
Other/multiracial^*b*^	20 (12.0)	11 (13.3)	10 (12.0)
Missing	1 (0.6)	1 (1.2)	0
Hispanic
Yes	6 (3.6)	1 (1.2)	5 (6.0)
No	159 (95.8)	81 (97.6)	78 (94.0)
Missing	1 (0.6)	1 (1.2)	0
Annual income^*c*^
< $19,000	22 (13.3)	—	—
$20,000–$39,000	54 (32.5)	—	—
$40,000–$74,999	61 (36.8)	—	—
$75,000–$99,000	15 (9.0)	—	—
≥ $100,000	10 (6.0)	—	—
Missing	4 (2.4)	—	—
Employment
Employed for wages	99 (59.6)	46 (55.4)	53 (63.8)
Self-employed	10 (6.0)	4 (4.8)	6 (7.2)
Homemaker	10 (6.0)	0	10 (12.1)
Student	44 (26.5)	31 (37.4)	13 (15.7)
Unemployed/other^*d*^	2 (1.2)	1 (1.2)	1 (1.2)
Missing	1 (0.6)	1 (1.2)	0
Education
High School/GED	4 (2.4)	3 (3.6)	1 (1.2)
Some college (1–3 years)	60 (36.1)	33 (39.8)	27 (32.5)
College (> 4 years, graduate)	101 (60.8)	46 (55.4)	55 (66.3)
Missing	1 (0.6)	1 (1.2)	0
Smoking
Never	149 (89.8)	70 (84.3)	79 (95.2)
Former	10 (6.0)	7 (8.4)	3 (3.6)
Current	5 (3.0)	4 (4.8)	1 (1.2)
Missing	2 (1.2)	2 (2.4)	0
Abbreviations: BMI, body mass index; GED, General Equivalency Diploma. ^***a***^Weight (kg)/height (m)^2^ (measured at enrollment). ^***b***^Includes Asian, Black/African American, Pacific Islander, American Indian/Alaskan Native. ^***c***^U.S. dollars, combined household income for both partners. ^***d***^Includes out of work, retired, unable to work.			

Urinary BPA concentrations were measured in 2,632 urine samples collected from 166 participants (83 male, 83 female). Of the 2,632 urine samples, 18 were excluded due to inadequate labeling, leaving 2,614 samples for analysis (Table 2). The majority (*n* = 2,498, 95.6%) of samples were first-morning samples, and the remaining 116 (4.4%) were spot samples collected later in the day when the participant forgot to collect upon waking.

**Table 2:** Distribution of urinary BPA concentrations (ng/mL) and intraclass correlation coefficients among 83 males and 83 females.

Sample type	*n*	*n* > LOQ (%)	Minimum	Percentile					Maximum	GM (95% CI)	ICC (95% CI)
				5th	25th	50th	75th	95th			
All subjects	2,614	2,318 (88.7)	< LOQ	< LOQ	1.27	2.40	4.59	14.8	160	2.52 (2.27, 2.79)	0.15 (0.11, 0.19)
Male	618	558 (90.3)	< LOQ	< LOQ	1.43	2.64	4.94	17.5	92.9	2.78 (2.39, 3.22)	0.18 (0.11, 0.26)
Female	1,996	1,760 (88.2)	< LOQ	< LOQ	1.22	2.33	4.44	14.2	160	2.44 (2.15, 2.77)	0.11 (0.08, 0.16)
First-morning urine	2,498	2,218 (88.8)	< LOQ	< LOQ	1.28	2.40	4.61	15.1	160	2.53 (2.28, 2.81)	0.15 (0.11, 0.20)
Male	574	520 (90.6)	< LOQ	< LOQ	1.44	2.65	5.10	17.7	92.9	2.81 (2.41, 3.29)	0.18 (0.11, 0.26)
Female	1,924	1,698 (88.3)	< LOQ	< LOQ	1.24	2.36	4.48	14.2	160	2.45 (2.15, 2.78)	0.11 (0.08, 0.16)
Spot urine	116	100 (86.2)	< LOQ	< LOQ	1.04	2.17	4.40	13.8	47.2	2.27 (1.86, 2.78)	—
Male	44	38 (86.4)	< LOQ	< LOQ	1.25	2.53	4.37	9.02	20.4	2.32 (1.69, 3.18)	—
Female	72	62 (86.1)	< LOQ	< LOQ	1.04	1.93	4.40	22.3	47.2	2.25 (1.73, 2.93)	—
Abbreviations: ICC, intraclass correlation coefficient; LOQ, limit of quantification. The LOQ was 0.75 ng/mL and values < LOQ were replaced with LOQ divided by the square root of 2 or 0.53 ng/mL.											

Among the 2,614 urine samples, 1,996 were collected by females and 618 were collected by males (Table 2). On average, females collected 17 ± 5 samples per person/cycle (range, 1–24), and males collected during the female partner’s fertile window with an average of 6 ± 2 samples per person/cycle (range, 1–12). Among females, 49 (59%) collected samples for two menstrual cycles, and 47 (56.6%) males collected during the fertile window for two partner menstrual cycles (two men failed to collect samples when their partner was collecting). Twenty-four couples became pregnant during the first cycle of sample collection, and 10 couples became pregnant during the second cycle of sample collection. The median BPA concentration was 2.40 ng/mL among all individual samples, with a range from < LOQ to 160.0 ng/mL. The GM for the entire cohort was 2.52 ng/mL (95% CI: 2.27, 2.79). Males had slightly higher median values (2.64 ng/mL) than did females (2.33 ng/mL) as well as a higher GM (males: 2.78 ng/mL; 95% CI: 2.39, 3.22, females: 2.44 ng/mL; 95% CI: 2.15, 2.77). The ICC for all samples was 0.15 (95% CI: 0.11, 0.19), indicating high within-person variation. Males had a slightly higher ICC (0.18, 95% CI: 0.11, 0.26) compared with the overall and compared with females (0.11, 95% CI: 0.08, 0.16). Limiting the calculation of ICC to first-morning samples and to individual cycles did not substantially change these results.

**Figure 1:**
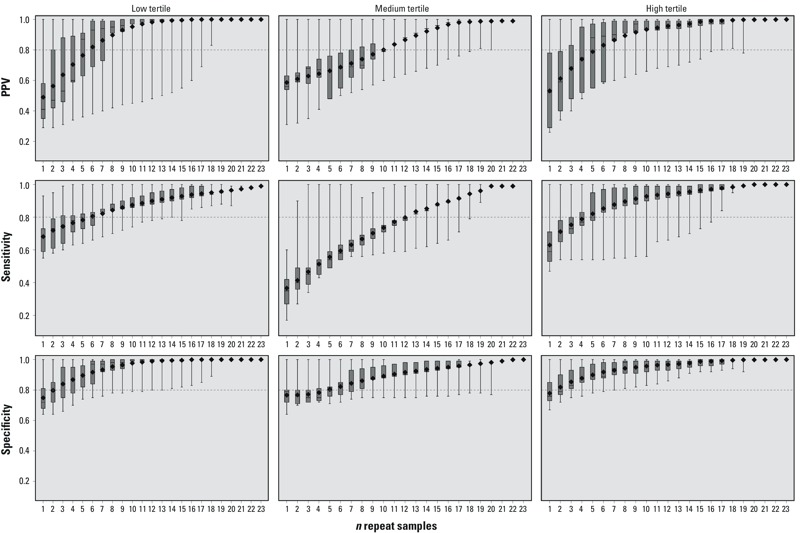
Surrogate category analysis among females, analysis 1, for PPV, sensitivity, and specificity. The displayed values are the minimum (bottom whisker), 25th percentile (bottom of box), median (line in box), mean (diamond), 75th percentile (top of box), and maximum (top whisker). The horizontal line at 0.8 is a reference line. The *x*-axis represents the number of repeated samples. Tertiles were defined as follows: low (< 1.76 ng/mL), medium (≥ 1.76 ng/mL to < 3.33 ng/mL), high (≥ 3.33 ng/mL). For corresponding numeric data, see Supplemental Material, Table S1.

*Exposure classification analysis.* Female, analysis 1. Statistics (sensitivity, specificity, and PPV) summarizing the ability of repeat samples to correctly predict a participant’s “true” exposure classification are shown in Figure 1 (see Supplemental Material, Table S1, for corresponding numeric data). Exposure tertiles for females were defined as follows based on the distribution among all female samples: low (< 1.76 ng/mL), medium (≥ 1.76 ng/mL to < 3.33 ng/mL), high (≥ 3.33 ng/mL). The median proportion of females who “truly” were in the highest exposure tertile and were classified as such based on 1 randomly selected sample (i.e., sensitivity) was 0.59 [interquartile range (IQR), 0.53–0.71] but increased with additional samples and reached 0.80 (IQR, 0.78–0.95) when at least 5 samples were included. Similarly, 5 repeat samples were required to reach a median sensitivity of 0.80 (IQR, 0.73–0.99) for females within the lowest tertile of exposure. Sensitivity to correctly categorize females in the medium tertile was quite a bit lower compared to the high and low tertiles, with median sensitivity of only 0.35 (IQR, 0.27–0.42) and 0.54 (IQR, 0.49–0.59) for 1 and 5 samples, respectively, and 13 samples required to reach 80% sensitivity.

Five repeat samples were required for females within the high tertile of BPA exposure to exceed a proportion of 0.80 correctly classified into the high tertile when they belonged in that tertile based on the GM of their samples from a given cycle (i.e., PPV) (PPV = 0.88; IQR, 0.55–0.97). When only a single sample was used to classify in the high tertile, PPV was 0.52 (IQR, 0.29–0.78). Classification for females truly in the low tertile was similar and required five repeat samples to reach a PPV of 0.87 (IQR, 0.63–0.91).

Specificity—the proportion of participants who were not classified into a tertile when they were “truly” not in that tertile—was higher than sensitivity and PPV, and only three samples were required to reach or exceed a proportion of 0.80 for specificity in the high and low tertiles, but five were required for the medium tertile.

**Figure 2:**
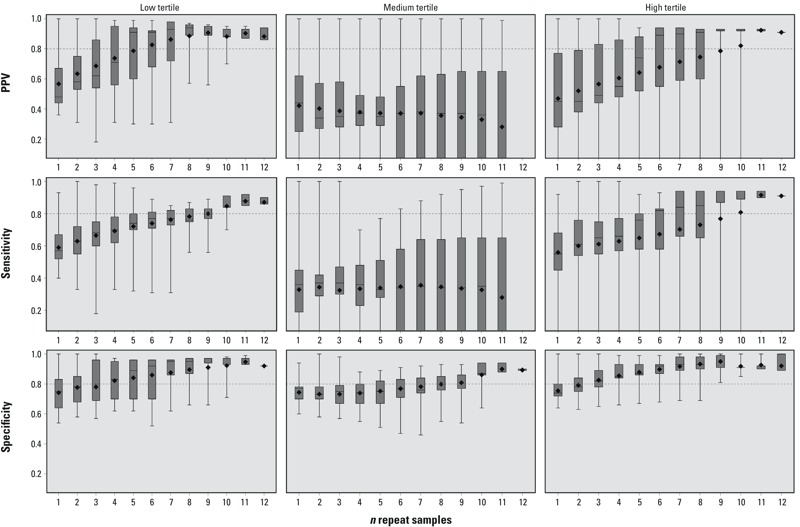
Surrogate category analysis among females, analysis 2, for PPV, sensitivity, and specificity. The displayed values are the minimum (bottom whisker), 25th percentile (bottom of box), median (line in box), mean (diamond), 75th percentile (top of box), and maximum (top whisker). The horizontal line at 0.8 is a reference line. The *x-*axis represents the number of repeated samples. Tertiles were defined as follows: low (< 1.76 ng/mL), medium (≥ 1.76 ng/mL to < 3.33 ng/mL), high (≥ 3.33 ng/mL). For corresponding numeric data, see Supplemental Material, Table S2.

Female, analysis 2. When the repeat samples used in the surrogate tertile were not used in the calculation of the “true” GM, the sensitivity, specificity, and PPV dropped compared with those of analysis 1 (Figure 2; for corresponding numeric data, see Supplemental Material, Table S2). When only 1 sample was used, the sensitivity in all three exposure tertiles had medians < 0.60 (low, 0.57; medium, 0.36; high, 0.55). Six repeat samples were sufficient to exceed a median proportion of 0.80 for both sensitivity and PPV in the high exposure tertile. Specificity remained much the same as in analysis 1, and 3 samples correctly classified both the low and high tertiles with a median proportion > 0.80. Even with 11 repeat samples, median sensitivity and PPV in the medium tertile was ≤ 0.36. This is likely attributable to participants’ having samples with a mixture of both high and low BPA concentrations, which makes categorization less consistent for the medium tertile, although specificity is 0.88 (95% CI: 0.88, 0.94) with 11 samples.

**Figure 3:**
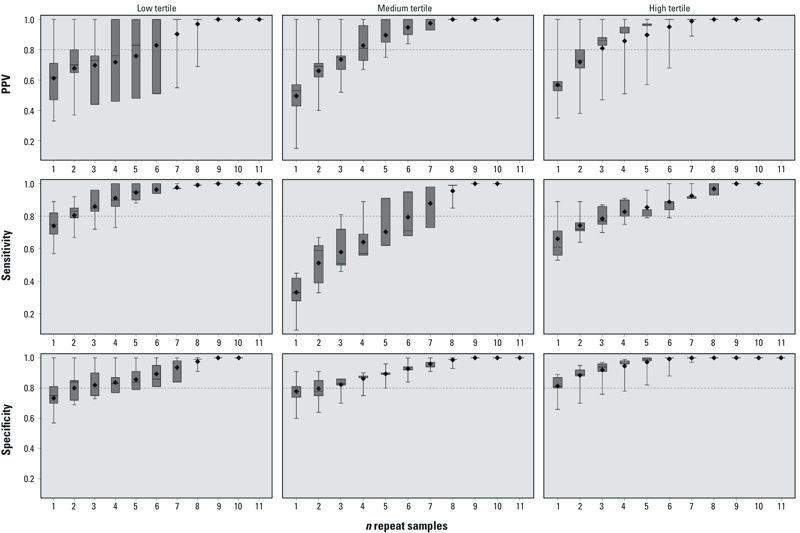
Surrogate category analysis among males, analysis 1, for PPV, sensitivity, and specificity. The displayed values are the minimum (bottom whisker), 25th percentile (bottom of box), median (line in box), mean (diamond), 75th percentile (top of box), and maximum (top whisker). The horizontal line at 0.8 is a reference line. The *x*-axis represents the number of repeated samples. Tertiles were defined as follows: low (< 2.20 ng/mL), medium (≥ 2.20 ng/mL to < 4.15 ng/mL), high (≥ 4.15 ng/mL). For corresponding numeric data, see Supplemental Material, Table S3.

Male, analysis 1. As described earlier, fewer samples were available from males for analysis. Exposure tertiles for males were defined as follows: low (< 2.20 ng/mL), medium (≥ 2.20 ng/mL to < 4.15 ng/mL), high (≥ 4.15 ng/mL). Four repeat samples were sufficient for males to reach a median sensitivity of 0.80 in the high tertile (0.80; IQR, 0.80–0.90), and two samples were needed to exceed 0.80 for the low tertile (0.83; IQR, 0.79–0.85) (Figure 3; for corresponding numeric data, see Supplemental Material, Table S3). Three repeat samples generated a median PPV for the high tertile of 0.86 (IQR, 0.83–0.88) and 0.73 (IQR, 0.44–0.76) for the low tertile. Similar to the patterns seen in females, males had higher and more precise values for specificity, with only one to two samples required to exceed a median proportion of 0.80 in each of the three tertiles of exposure, and multiple samples were less accurate for classifying sensitivity and PPV in the medium tertile.

**Figure 4:**
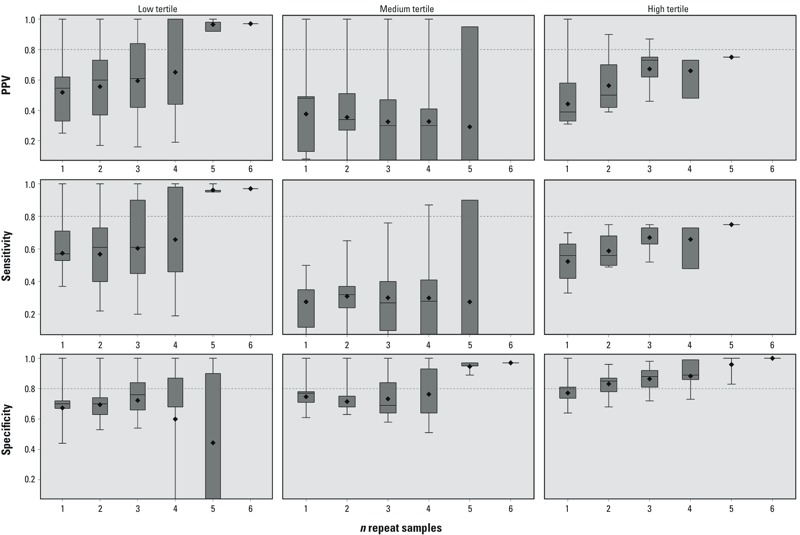
Surrogate category analysis among males, analysis 2, for PPV, sensitivity, and specificity. The displayed values are the minimum (bottom whisker), 25th percentile (bottom of box), median (line in box), mean (diamond), 75th percentile (top of box), and maximum (top whisker). The horizontal line at 0.8 is a reference line. The *x*-axis represents the number of repeated samples. Tertiles were defined as follows: low (< 2.20 ng/mL), medium (≥ 2.20 ng/mL to < 4.15 ng/mL), high (≥ 4.15 ng/mL). For corresponding numeric data, see Supplemental Material, Table S4.

Male, analysis 2. When a single sample was used to predict the high exposure tertile, the median sensitivity and PPV were 0.56 (IQR, 0.42–0.63) and 0.39 (IQR, 0.33–0.58), respectively (Figure 4; for corresponding numeric data, see Supplemental Material, Table S4). These both increased with additional samples and reached a maximum of 0.75 with five repeat samples. The median sensitivity with a single sample to predict the low exposure tertile was 0.57 (IQR, 0.53–0.71), and this increased to 0.95 (IQR, 0.95–0.96) when five repeat samples were used. The PPV for the low tertile with five repeat samples was similar (PPV = 0.98; IQR, 0.92–0.98).

## Discussion

The urinary BPA GM concentrations among HOPE study females was higher than that reported in other studies which have reported unadjusted or specific gravity–adjusted GMs < 2.0 μg/L among pregnant women with 1–5 samples per woman in Canada (Fisher et al. 2015), the Netherlands (Jusko et al. 2014), New York City (Perera et al. 2012), Mexico City (Cantonwine et al. 2010), California (Harley et al. 2013), and in the National Children’s Study (Mortensen et al. 2014). The median concentration among HOPE females (2.33 ng/mL) is similar to other unadjusted or specific gravity–adjusted medians with 1–5 samples per woman reported in Boston, Massachusetts (Braun et al. 2012; Mahalingaiah et al. 2008), Puerto Rico (Meeker et al. 2013), France (Philippat et al. 2012), and Spain (Casas et al. 2011). In part, differences in concentrations may occur because most HOPE samples were collected as first-morning voids, which have been shown to yield higher BPA concentrations than do spot and 24-hr collection (Christensen et al. 2012). This may also be partly attributable to the fact that females in the HOPE study collected a mean 17 ± 5 samples per cycle compared with other studies with a single spot sample or set of 3–5 spot samples per female, usually collected over the trimesters of pregnancy and postpartum. When comparing HOPE study results (where some women became pregnant during specimen collection but some did not) with studies of pregnant women, it is important to note that pregnancy status is likely to influence exposure patterns and physiology, and may affect urinary concentrations and variability, making direct comparison of pregnant and non-pregnant women warrant caution.

Among HOPE participant males, concentrations were also higher than those in other studies reporting unadjusted geometric means or medians < 2.0 μg/L in Michigan and Texas (Goldstone et al. 2015), California (Mendiola et al. 2010), Boston (Mahalingaiah et al. 2008), and Denmark (Lassen et al. 2014), although another study among 308 Danish men reports an unadjusted median concentration of 3.25 ng/mL (Lassen et al. 2013).

We observed high within-person variability relative to total variability of serial urinary BPA samples over one to two menstrual cycles, with an ICC of 0.11 among females and 0.18 among males. Among females, this is consistent with or lower than findings from 389 pregnant women in Ohio with three spot samples collected during pregnancy (ICC = 0.10–0.12 creatinine adjusted, ICC = 0.25–0.28 unadjusted) (Braun et al. 2011), female nurses in 14 states with two first morning samples collected 1–3 years apart (ICC = 0.14) (Townsend et al. 2013), 105 women in Puerto Rico with three samples collected during pregnancy (ICC = 0.24–0.27) (Meeker et al. 2013), 137 women in Boston with two specimens collected before pregnancy and three collected during pregnancy (ICC = 0.23 pre-pregnancy, ICC = 0.12 during pregnancy) (Braun et al. 2012), and among women trying to conceive with three repeat samples in a single menstrual cycle in North Carolina (ICC = 0.43) (Nepomnaschy et al. 2009). Our ICC was slightly higher than that observed by Fisher et al. (2015) (ICC = 0.07), who measured at five time points across pregnancy and postpartum. Among this analytical subset of HOPE couples, 34 became pregnant during specimen collection (24 in cycle 1 and 10 in cycle 2). Within a single cycle for women who conceived, the first few specimens were collected when she was not pregnant, but the remainder of the cycle’s samples were collected when she was pregnant. The exact number of pregnant versus non-pregnant days for each cycle cannot be calculated without knowing when implantation occurred, but this change in the woman’s physiology could be a source of additional within-person variability during a given cycle. Lassen et al. (2013) examined reproducibility over a 3-month period in spot, first-morning, and 24-hr urine samples among males and found unadjusted ICC values of 0.42, 0.10, and 0.26, respectively. Our observed ICC for first-morning samples among males is similar but slightly higher (ICC = 0.18).

The sensitivity of a single sample, among females, to predict the high exposure tertile observed in this study (analysis 1: 0.59; IQR, 0.53–0.71) was slightly lower than the sensitivity observed by both Braun et al. (2012) (0.60–0.70) and Mahalingaiah et al. (2008) (0.64), whose methods were similar to those used in analysis 1 of our surrogate category analysis (Table 3). PPV and specificity were similarly low compared to Braun et al. (2012) and Mahalingaiah et al. (2008). Fisher et al. (2015) also performed a surrogate category analysis that excluded the surrogate sample from the “true” GM (similar to analysis 2 with slightly different methods) and observed sensitivity and PPV for a single sample that was higher (0.61–0.65) than observed in analysis 2 of this study among females, although their analysis was based on different sample collection methods with fewer samples per person (two 24-hr samples and three spot samples), and all participants were pregnant. We consider the analysis 2 analysis among females to be the most precise in this study for two reasons: The high number of samples per person lends itself to a more accurate characterization of typical exposure, and the inherent structural dependency was removed through analytic methods. We believe that sensitivity, specificity, and PPV were overinflated when the sample used to calculate the surrogate GM was also used to calculate the overall GM, given that results for analysis 2 of the surrogate category analysis were generally lower compared with analysis 1. Males consistently required fewer repeat samples for correct categorization compared to females, but this is likely attributable to the fact that we had fewer total samples for analysis for males than for females, and samples were collected over a shorter period of time, which could contribute to less variation.

**Table 3:** Surrogate category analysis study comparison table.

Study	Study population	BPA exposure characterization	*n* samples^*a*^	Sensitivity	Specificity	PPV
HOPE study, analysis 1^*b*^^,^^*c*^	83 women (subset of HOPE study)	Serial daily first-morning collection during pre/conception cycles	1 2	0.59 0.65	0.76 0.79	0.52 0.59
HOPE study, analysis 2^*b*^^,^^*d*^	Same as above	Same as above	1 2	0.55 0.61	0.74 0.78	0.45 0.45
Braun et al. 2012	91 women from subset of EARTH study	3 or more spot samples collected from preconception to delivery	1	1st trimester, 0.70, 2nd trimester, 0.60, 3rd trimester, 0.67	1st trimester, 0.85, 2nd trimester, 0.80, 3rd trimester, 0.84	1st trimester, 0.70, 2nd trimester, 0.60, 3rd trimester, 0.67
Mahalingaiah et al. 2008^*e*^	Couples seeking infertility treatment in Boston	149 samples from 31 subjects with at least 3 repeat samples^*f*^	1	0.64	0.76	0.63
Mahalingaiah et al. 2008^*e*^	Same as above	67 samples from 8 subjects with at least 6 repeat samples^*f*^	2	0.67	0.84	0.85
Fisher et al. 2015^*g*^	80 pregnant women (P4 Study)	5 repeat samples from early pregnancy to delivery^*h*^	1	0.65	0.66	0.61
Abbreviations: BPA, bisphenol A; PPV, positive predictive value. ^***a***^Number of samples used to calculate predictive statistics (sensitivity, specificity, and PPV). ^***b***^Results for 1 and 2 repeat samples to predict high tertile among females presented for comparison. ^***c***^Methods most directly comparable to those of Braun et al. (2012) and Mahalingaiah et al. (2008). ^***d***^Methods most directly comparable to those of Fisher et al. (2015). ^***e***^Men and women included in analyses together. ^***f***^Spot samples collected at recruitment, at clinic visits during treatment, and during post-treatment clinical appointments. Women also collected samples during the trimesters of pregnancy. ^***g***^Statistics calculated with slightly different methods. ^***h***^Two samples were 24-hr collected before 20 weeks gestation, and three were spot samples collected during the second and third trimesters and then 2–3 months postpartum.						

One strength of this study is the high number of samples per person relative to previous studies, which may result in a more accurate categorization of the individual’s “true” exposure—assuming that a GM of a higher number of repeated samples is a more accurate assessment of the individual’s level of exposure over time. The predominance of highly concentrated first-morning samples and high compliance with sample collection also strengthens the exposure assessment in this study. This study is also unique in that it considers both males and females separately; it has been observed that males and females have different patterns of exposure with regard to BPA, and BPA may exert potential effects differently between males and females (Lakind et al. 2014). However, our ability to draw conclusions about differences between males and females is limited by differences in the number of samples and sampling duration.

Although this study predicts only one cycle of exposure at a time, BPA concentrations between cycles were correlated (Spearman correlation ρ = 0.67, *p* < 0.001 for females, ρ = 0.64, *p* < 0.001 for males). This moderate correlation is higher than that observed in samples collected among pregnant women (Braun et al. 2011, 2012; Fisher et al. 2015; Jusko et al. 2014), although these studies collected single spot samples spread over weeks to months. It is possible that we observed higher correlation because we had more samples per person, and time between sample collections was reduced. Nepomnaschy et al. (2009) also observed moderate Spearman correlations of ρ = 0.53 and ρ = 0.56 with samples collected 14 days apart and ρ = 0.30 for the total time separation of 28 days among women trying to conceive. We believe the combination of the accurate assessment of daily exposure from repeated sampling in this study with the fact that cycles are correlated allowed us to conclude that the assigned tertile categorization would be consistent over time and reflect the individual’s likely habitual and long-term exposure.

Generalizability of our study may be limited by the proportion of highly educated, high-income Caucasian individuals in the cohort. These characteristics can affect measured urinary concentrations of BPA through differences in exposures related to diet, employment, or other socioeconomic factors (Calafat et al. 2008). This group of participants was also highly motivated and collected specimens with high compliance to the study protocol; thus, this urine collection protocol may not be possible in other populations that are less motivated, and it may not be reasonable to expect such a high number of samples per person in other studies.

Previous studies have also reported that time of day, fasting time, and time since last void may affect urinary BPA concentrations (Braun et al. 2011; Fisher et al. 2015; Mahalingaiah et al. 2008; Ye et al. 2011), but Stahlhut et al. (2009) did not find a strong association between fasting time and BPA. Therefore, it may be a limitation that daily exposure, fasting time, time of sample collection, and time since previous void are not available in our data and could not be examined in our analyses. It may also be a limitation that exposure classification was performed on first-morning voids that could be inherently different from other voids throughout the day and in 24-hr samples. First-morning voids are likely to reflect exposure that occurred during dinner the night before but may not accurately reflect exposure that took place during the earlier hours of the day. However, Christensen et al. (2012) found that the distributions of 24-hr and subsequent first-morning voids from the same individual were similar (Cramer–von Mises criterion values 0.0002 and 0.002, respectively) with similar variance (Levene’s *p*-values 0.02 and 0.003, respectively), and Lassen et al. (2013) found that 24-hr and subsequent first-morning voids were moderately correlated (Spearman ρ = 0.56, *p* < 0.001), although Ye et al. (2011) concluded that first-morning voids were not a good surrogate for 24-hr samples. In future studies one could compare the classification with repeat samples such as we have done here with 24-hr samples from the same individual.

## Conclusion

Urinary BPA concentrations have a high degree of variability both within and between individuals. Evidence from this and other studies suggests that characterization of BPA exposure through a single, or even 2 repeat samples, may result in substantial misclassification and subsequent attenuation of effect estimates. When considering study design and sampling strategy, the purpose of the assessment must be considered. If long-term characterization of exposure is desired, repeated sampling is required, but in cases where repeat sampling is not feasible or fewer samples are collected, our results may be used by the researcher to estimate the predictive power of the number of collected samples. Based on our more stringent surrogate category analysis 2, to reach proportions ≥ 0.80 for sensitivity, specificity, and PPV among females, 6 and 10 repeat samples for the high and low tertiles, respectively, were required. For the medium tertile, specificity reached 0.87 with 10 repeat samples, but even with 11 samples, sensitivity and PPV did not exceed 0.36. Five repeat samples, among males, yielded sensitivity and PPV values ≥ 0.75 for the high and low tertiles, but, as with females, classification for the medium tertile was less accurate. Our results suggest that individuals who would typically be classified into the medium tertile of exposure likely have combinations of high and low exposures that make classification into tertiles without repeated sampling, analyses of temporal variability, and analyses of exposure classification more complicated and less accurate. These results are based on daily, repeat sampling of first-morning urine that is uncorrected for urinary dilution, in a menstrual cycle (female) and a fertile window (male). Spot samples or samples collected over a greater period of time may be more variable.

## Supplemental Material

(399 KB) PDF
