# Supplementary material for: Exposure Classification and Temporal Variability in Urinary Bisphenol A Concentrations among Couples in Utah—The HOPE Study
Source: Environ Health Perspect. 2015 Sep 15;124(4):498–506. doi: 10.1289/ehp.1509752 (PMC4829981; doi:10.1289/ehp.1509752)
Supplement: (399 KB) PDF [file ehp.1509752.s001.acco.pdf]

Environ Health Perspect DOI: 10.1289/ehp.1509752
Advance Publication: Not Copyedited

Note to Readers: *EHP* strives to ensure that all journal content is accessible to all readers. However, some figures and Supplemental Material published in *EHP* articles may not conform to 508 standards due to the complexity of the information being presented. If you need assistance accessing journal content, please contact ehp508@niehs.nih.gov. Our staff will work with you to assess and meet your accessibility needs within 3 working days.

Supplemental Material

Exposure Classification and Temporal Variability in Urinary Bisphenol-A Concentrations among Couples in Utah—The HOPE Study

Kyley J. Cox, Christina A. Porucznik, David J. Anderson, Eric M. Brozek, Kathryn M. Szczotka, Nicole M. Bailey, Diana G. Wilkins, and Joseph B. Stanford

Table of Contents

Table S1. Surrogate Category Analysis among Females, Analysis 1
Table S2. Surrogate Category Analysis among Females, Analysis 2
Table S3. Surrogate Category Analysis among Males, Analysis 1
Table S4. Surrogate Category Analysis among Males, Analysis 2

Table S1. Surrogate category analysis among females, Analysis 1

Repeat samples	Sensitivity		Specificity		Positive Predictive Value	
	Median (IQR)	Mean (95% CI)	Median (IQR)	Mean & CI	Median (IQR)	Mean & CI
High Tertile						
1	0.59 (0.53, 0.71)	0.63 (0.62, 0.64)	0.76 (0.73, 0.85)	0.78 (0.78, 0.78)	0.52 (0.29, 0.78)	0.53 (0.52, 0.54)
2	0.65 (0.65, 0.78)	0.71 (0.71, 0.71)	0.79 (0.77, 0.90)	**0.82 (0.82, 0.82)**	0.59 (0.40, 0.79)	0.61 (0.61, 0.62)
3	0.73 (0.70, 0.80)	0.75 (0.75, 0.75)	**0.83 (0.81, 0.93)**	0.85 (0.85, 0.85)	0.69 (0.48, 0.83)	0.68 (0.68, 0.68)
4	0.77 (0.75, 0.83)	0.79 (0.79, 0.79)	0.85 (0.85, 0.95)	0.88 (0.88, 0.88)	0.75 (0.52, 0.95)	0.74 (0.74, 0.74)
5	**0.80 (0.78, 0.95)**	**0.82 (0.82, 0.82)**	0.87 (0.87, 0.97)	0.90 (0.90, 0.90)	**0.88 (0.55, 0.97)**	0.79 (0.79, 0.79)
6	0.83 (0.83, 0.97)	0.85 (0.85, 0.85)	0.89 (0.88, 0.98)	0.92 (0.92, 0.92)	0.89 (0.59, 0.98)	**0.83 (0.83, 0.83)**
7	0.85 (0.85, 0.98)	0.88 (0.88, 0.88)	0.94 (0.90, 0.98)	0.93 (0.93, 0.94)	0.90 (0.86, 0.99)	0.87 (0.86, 0.87)
8	0.87 (0.87, 0.98)	0.90 (0.90, 0.90)	0.98 (0.91, 0.99)	0.94 (0.94, 0.95)	0.91 (0.91, 0.99)	0.89 (0.89, 0.89)
9	0.88 (0.88, 0.99)	0.91 (0.91, 0.91)	0.99 (0.91, 0.99)	0.95 (0.95, 0.96)	0.97 (0.92, 0.99)	0.92 (0.92, 0.92)
10	0.90 (0.90, 0.99)	0.93 (0.93, 0.93)	0.99 (0.92, 0.99)	0.96 (0.96, 0.96)	0.99 (0.93, 0.99)	0.93 (0.93, 0.93)
11	0.91 (0.91, 0.99)	0.94 (0.94, 0.94)	0.99 (0.93, 0.99)	0.96 (0.96, 0.97)	0.99 (0.93, 0.99)	0.95 (0.95, 0.95)
12	0.92 (0.92, 0.99)	0.94 (0.94, 0.94)	0.99 (0.93, 0.99)	0.97 (0.97, 0.97)	0.99 (0.94, 0.99)	0.96 (0.96, 0.96)
13	0.93 (0.93, 0.99)	0.95 (0.95, 0.95)	0.99 (0.94, 0.99)	0.97 (0.97, 0.98)	0.99 (0.94, 0.99)	0.96 (0.96, 0.96)
14	0.94 (0.94, 0.99)	0.96 (0.96, 0.96)	0.99 (0.95, 0.99)	0.98 (0.98, 0.99)	0.99 (0.95, 0.99)	0.97 (0.97, 0.97)
15	0.95 (0.95, 1.00)	0.97 (0.97, 0.97)	1.00 (0.96, 1.00)	0.99 (0.99, 0.99)	1.00 (0.96, 1.00)	0.98 (0.98, 0.98)
16	0.96 (0.96, 1.00)	0.97 (0.97, 0.97)	1.00 (0.96, 1.00)	0.99 (0.99, 0.99)	1.00 (0.97, 1.00)	0.99 (0.99, 0.99)
17	0.97 (0.97, 1.00)	0.98 (0.98, 0.98)	1.00 (0.97, 1.00)	0.99 (0.99, 0.99)	1.00 (0.97, 1.00)	0.99 (0.99, 0.99)
18	0.98 (0.98, 0.98)	0.98 (0.98, 0.98)	1.00 (1.00, 1.00)	1.00 (1.00, 1.00)	1.00 (1.00, 1.00)	1.00 (1.00, 1.00)
19	0.99 (0.99, 0.99)	0.99 (0.99, 0.99)	1.00 (1.00, 1.00)	1.00 (1.00, 1.00)	1.00 (1.00, 1.00)	1.00 (1.00, 1.00)
20-23	1.00 (1.00, 1.00)	1.00 (1.00, 1.00)	1.00 (1.00, 1.00)	1.00 (1.00, 1.00)	1.00 (1.00, 1.00)	1.00 (1.00, 1.00)
Medium Tertile						
1	0.35 (0.27, 0.42)	0.37 (0.36, 0.37)	0.79 (0.72, 0.80)	0.77 (0.76,0.77)	0.56 (0.54, 0.63)	0.59 (0.58, 0.59)
2	0.40 (0.36, 0.49)	0.41 (0.41, 0.42)	0.76 (0.71, 0.80)	0.77 (0.77,0.77)	0.63 (0.59, 0.65)	0.61 (0.61, 0.61)
3	0.45 (0.39, 0.49)	0.47 (0.47, 0.47)	0.75 (0.72, 0.80)	0.77 (0.77,0.77)	0.68 (0.58, 0.69)	0.63 (0.63, 0.63)
4	0.51 (0.45, 0.54)	0.51 (0.51, 0.51)	0.75 (0.73, 0.81)	0.78 (0.78,0.78)	0.67 (0.62, 0.74)	0.64 (0.64, 0.65)
5	0.54 (0.49, 0.59)	0.56 (0.56, 0.56)	**0.82 (0.76, 0.82)**	**0.80 (0.80,0.80)**	0.66 (0.48, 0.76)	0.66 (0.66, 0.66)
6	0.58 (0.54, 0.63)	0.59 (0.59, 0.59)	0.84 (0.77, 0.85)	0.82 (0.82,0.82)	0.68 (0.55, 0.78)	0.69 (0.69, 0.69)
7	0.60 (0.59, 0.66)	0.63 (0.63, 0.63)	0.85 (0.79, 0.93)	0.84 (0.84,0.84)	0.69 (0.62, 0.80)	0.71 (0.71, 0.71)
8	0.63 (0.63, 0.69)	0.67 (0.67, 0.67)	0.86 (0.81, 0.94)	0.86 (0.86,0.86)	0.70 (0.68, 0.82)	0.74 (0.74, 0.74)
9	0.67 (0.67, 0.73)	0.70 (0.70, 0.70)	0.87 (0.87, 0.95)	0.88 (0.88,0.88)	0.74 (0.74, 0.84)	0.77 (0.77, 0.77)
10	0.71 (0.71, 0.75)	0.74 (0.74, 0.74)	0.88 (0.88, 0.96)	0.89 (0.89,0.89)	0.79 (0.79, 0.79)	**0.80 (0.80, 0.80)**
11	0.75 (0.75, 0.78)	0.77 (0.77, 0.77)	0.89 (0.89, 0.97)	0.90 (0.90,0.90)	**0.84 (0.84, 0.84)**	0.84 (0.84, 0.84)
12	0.78 (0.78, 0.80)	**0.80 (0.80, 0.80)**	0.90 (0.90, 0.98)	0.92 (0.92,0.92)	0.88 (0.88, 0.88)	0.87 (0.87, 0.87)
13	**0.82 (0.82, 0.82)**	0.83 (0.83, 0.83)	0.91 (0.91, 0.98)	0.92 (0.92,0.92)	0.91 (0.91, 0.91)	0.89 (0.89, 0.89)
14	0.84 (0.84, 0.84)	0.85 (0.85, 0.85)	0.92 (0.92, 0.99)	0.94 (0.94,0.94)	0.94 (0.94, 0.94)	0.92 (0.92, 0.92)
15	0.87 (0.87, 0.87)	0.88 (0.88, 0.88)	0.93 (0.93, 0.99)	0.94 (0.94,0.94)	0.96 (0.96, 0.96)	0.94 (0.94, 0.94)
16	0.89 (0.89, 0.89)	0.90 (0.90, 0.90)	0.94 (0.94, 0.99)	0.95 (0.95,0.95)	0.98 (0.98, 0.98)	0.97 (0.97, 0.97)
17	0.91 (0.91, 0.91)	0.92 (0.92, 0.92)	0.95 (0.95, 0.99)	0.96 (0.96,0.96)	0.99 (0.99, 0.99)	0.98 (0.98, 0.98)
18	0.94 (0.94, 0.94)	0.94 (0.94, 0.94)	0.96 (0.96, 0.96)	0.97 (0.97,0.97)	0.99 (0.99, 0.99)	0.98 (0.98, 0.98)
19	0.96 (0.96, 0.96)	0.96 (0.96, 0.96)	0.97 (0.97, 0.97)	0.97 (0.97,0.97)	0.99 (0.99, 0.99)	0.99 (0.99, 0.99)
20	0.99 (0.99, 0.99)	0.99 (0.99, 0.99)	0.98 (0.98, 0.98)	0.98 (0.98,0.98)	0.99 (0.99, 0.99)	0.99 (0.99, 0.99)
21-23	0.99 (0.99, 0.99)	0.99 (0.99, 0.99)	0.99 (0.99, 0.99)	0.99 (0.99,0.99)	0.99 (0.99, 0.99)	0.99 (0.99, 0.99)
Low Tertile						
1	0.68 (0.59, 0.95)	0.68 (0.68,0.69)	0.72 (0.68, 0.81)	0.75 (0.75, 0.75)	0.41 (0.35, 0.58)	0.49 (0.48,0.50)
2	0.73 (0.61, 0.96)	0.72 (0.72,0.72)	0.79 (0.72, 0.85)	**0.80 (0.80, 0.80)**	0.47 (0.42, 0.80)	0.56 (0.56,0.57)
3	0.75 (0.64, 0.97)	0.74 (0.74,0.74)	**0.84 (0.75, 0.95)**	0.84 (0.84, 0.84)	0.53 (0.46, 0.88)	0.64 (0.64,0.64)
4	0.78 (0.71, 0.98)	0.77 (0.77,0.77)	0.87 (0.78, 0.95)	0.87 (0.87, 0.87)	0.60 (0.59, 0.89)	0.70 (0.70,0.71)
5	**0.80 (0.73, 0.99)**	0.78 (0.78,0.78)	0.90 (0.82, 0.96)	0.90 (0.90, 0.90)	**0.87 (0.63, 0.91)**	0.76 (0.76,0.77)
6	0.83 (0.75, 0.83)	**0.81 (0.81,0.81)**	0.97 (0.84, 0.99)	0.92 (0.92, 0.92)	0.93 (0.69, 0.99)	**0.82 (0.82,0.82)**

7	0.83 (0.83, 0.85)	0.82 (0.82,0.82)	0.97 (0.92, 0.99)	0.93 (0.93, 0.93)	0.94 (0.73, 0.99)	0.86 (0.86,0.86)
8	0.84 (0.84, 0.88)	0.84 (0.84,0.84)	0.98 (0.93, 1.00)	0.95 (0.95, 0.95)	0.95 (0.91, 0.99)	0.90 (0.90,0.90)
9	0.85 (0.85, 0.90)	0.86 (0.86,0.86)	0.98 (0.94, 1.00)	0.96 (0.96, 0.96)	0.96 (0.92, 1.00)	0.93 (0.93,0.93)
10	0.86 (0.86, 0.92)	0.87 (0.87,0.87)	0.99 (0.99, 1.00)	0.98 (0.98, 0.98)	0.97 (0.97, 1.00)	0.95 (0.95,0.95)
11	0.87 (0.87, 0.93)	0.89 (0.89,0.89)	0.99 (0.99, 1.00)	0.98 (0.98, 0.98)	1.00 (0.98, 1.00)	0.97 (0.97,0.97)
12	0.88 (0.88, 0.95)	0.90 (0.90,0.90)	1.00 (0.99, 1.00)	0.99 (0.99, 0.99)	1.00 (0.99, 1.00)	0.98 (0.98,0.98)
13	0.89 (0.89, 0.96)	0.91 (0.91,0.91)	1.00 (0.99, 1.00)	0.99 (0.99, 0.99)	1.00 (0.99, 1.00)	0.99 (0.99,0.99)
14	0.90 (0.90, .970)	0.92 (0.92,0.92)	1.00 (0.99, 1.00)	0.99 (0.99, 0.99)	1.00 (0.99, 1.00)	0.99 (0.99,0.99)
15	0.91 (0.91, 0.98)	0.93 (0.93,0.93)	1.00 (0.99, 1.00)	1.00 (1.00, 1.00)	1.00 (0.99, 1.00)	1.00 (1.00,1.00)
16	0.92 (0.92, 0.99)	0.94 (0.94,0.94)	1.00 (1.00, 1.00)	1.00 (1.00, 1.00)	1.00 (1.00, 1.00)	1.00 (1.00,1.00)
17	0.93 (0.93, 0.99)	0.94 (0.94,0.94)	1.00 (1.00, 1.00)	1.00 (1.00, 1.00)	1.00 (1.00, 1.00)	1.00 (1.00,1.00)
18	0.94 (0.94, 0.94)	0.95 (0.95,0.95)	1.00 (1.00, 1.00)	1.00 (1.00, 1.00)	1.00 (1.00, 1.00)	1.00 (1.00,1.00)
19	0.95 (0.95, 0.95)	0.96 (0.96,0.96)	1.00 (1.00, 1.00)	1.00 (1.00, 1.00)	1.00 (1.00, 1.00)	1.00 (1.00,1.00)
20	0.96 (0.96, 0.96)	0.96 (0.96,0.96)	1.00 (1.00, 1.00)	1.00 (1.00, 1.00)	1.00 (1.00, 1.00)	1.00 (1.00,1.00)
21	0.97 (0.97, 0.97)	0.97 (0.97,0.97)	1.00 (1.00, 1.00)	1.00 (1.00, 1.00)	1.00 (1.00, 1.00)	1.00 (1.00,1.00)
22	0.98 (0.98, 0.98)	0.98 (0.98,0.98)	1.00 (1.00, 1.00)	1.00 (1.00, 1.00)	1.00 (1.00, 1.00)	1.00 (1.00,1.00)
23	0.99 (0.99, 0.99)	0.99 (0.99,0.99)	1.00 (1.00, 1.00)	1.00 (1.00, 1.00)	1.00 (1.00, 1.00)	1.00 (1.00,1.00)

Abbreviations: IQR Interquartile Range, CI Confidence Interval

Corresponding figure in main text, Figure 1

Bold values represent the point at which 0.80 is met or exceeded in each category

Table S2. Surrogate category analysis among females, Analysis 2

Repeat samples	Sensitivity Median (IQR)	Sensitivity Mean (95% CI)	Specificity Median (IQR)	Specificity Mean & CI	Positive Predictive Value Median (IQR)	Positive Predictive Value Mean & CI
High Tertile						
1	0.55 (0.45, 0.68)	0.56 (0.55, 0.57)	0.74 (0.72, 0.8)	0.75 (0.75, 0.76)	0.45 (0.28, 0.77)	0.47 (0.46, 0.48)
2	0.61 (0.54, 0.76)	0.60 (0.60, 0.60)	0.78 (0.75, 0.84)	0.79 (0.79, 0.79)	0.45 (0.38, 0.79)	0.52 (0.52, 0.52)
3	0.65 (0.55, 0.75)	0.61 (0.61, 0.61)	**0.82 (0.78, 0.89)**	**0.83 (0.82, 0.83)**	0.49 (0.44, 0.83)	0.57 (0.49, 0.56)
4	0.66 (0.57, 0.77)	0.63 (0.63, 0.63)	0.85 (0.84, 0.93)	0.86 (0.85, 0.86)	0.55 (0.48, 0.86)	0.61 (0.55, 0.61)
5	0.76 (0.58, 0.80)	0.65 (0.65, 0.65)	0.87 (0.86, 0.93)	0.88 (0.88, 0.88)	0.74 (0.52, 0.88)	0.64 (0.64, 0.64)
6	**0.82 (0.58, 0.83)**	0.67 (0.67, 0.67)	0.92 (0.87, 0.93)	0.90 (0.90, 0.90)	**0.89 (0.55, 0.94)**	0.68 (0.68, 0.68)
7	0.84 (0.66, 0.94)	0.70 (0.70, 0.70)	0.93 (0.89, 0.98)	0.92 (0.92, 0.92)	0.90 (0.59, 0.94)	0.71 (0.71, 0.71)
8	0.85 (0.65, 0.94)	0.73 (0.73, 0.73)	0.93 (0.90, 0.98)	0.93 (0.93, 0.93)	0.91 (0.60, 0.93)	0.75 (0.75, 0.75)
9	0.87 (0.87, 0.94)	0.77 (0.77, 0.77)	0.99 (0.91, 0.99)	0.95 (0.95, 0.95)	0.92 (0.92, 0.93)	0.79 (0.79, 0.79)
10	0.89 (0.89, 0.94)	**0.81 (0.81, 0.81)**	0.91 (0.91, 0.91)	0.92 (0.92, 0.92)	0.92 (0.92, 0.93)	**0.82 (0.82, 0.82)**
11	0.90 (0.90, 0.94)	0.92 (0.92, 0.92)	0.92 (0.90, 0.92)	0.93 (0.93, 0.93)	0.92 (0.92, 0.93)	0.92 (0.92, 0.92)
12	0.91 (0.91, 0.91)	0.91 (0.91, 0.91)	0.89 (0.89, 1.00)	0.92 (0.92, 0.92)	0.91 (0.91, 0.91)	0.91 (0.91,0.91)
Medium Tertile						
1	0.36 (0.19, 0.45)	0.33 (0.32, 0.34)	0.77 (0.70, 0.78)	0.74 (0.74, 0.75)	0.44 (0.25, 0.62)	0.42 (0.41, 0.43)
2	0.37 (0.29, 0.42)	0.34 (0.34, 0.35)	0.73 (0.70, 0.78)	0.73 (0.73, 0.73)	0.34 (0.27, 0.57)	0.40 (0.40, 0.41)
3	0.37 (0.30, 0.47)	0.32 (0.32, 0.33)	0.75 (0.67, 0.79)	0.73 (0.73, 0.73)	0.35 (0.28, 0.58)	0.39 (0.38, 0.39)
4	0.36 (0.23, 0.48)	0.33 (0.33, 0.33)	0.74 (0.67, 0.80)	0.74 (0.74, 0.74)	0.37 (0.29, 0.49)	0.38 (0.38, 0.38)
5	0.33 (0.28, 0.51)	0.34 (0.34, 0.34)	0.75 (0.67, 0.82)	0.75 (0.75, 0.75)	0.35 (0.29, 0.48)	0.37 (0.37, 0.37)
6	0.34 (0.00, 0.58)	0.35 (0.36, 0.35)	0.77 (0.71, 0.83)	0.77 (0.77, 0.77)	0.37 (0.00, 0.55)	0.37 (0.37, 0.37)
7	0.35 (0.00, 0.64)	0.36 (0.36, 0.36)	0.76 (0.74, 0.84)	0.78 (0.78, 0.78)	0.38 (0.00, 0.62)	0.37 (0.37, 0.37)
8	0.34 (0.00, 0.64)	0.35 (0.35, 0.35)	0.76 (0.76, 0.85)	**0.80 (0.80, 0.80)**	0.37 (0.00, 0.63)	0.36 (0.36, 0.36)
9	0.34 (0.00, 0.65)	0.34 (0.34, 0.34)	0.77 (0.77, 0.86)	0.81 (0.81, 0.81)	0.37 (0.00, 0.65)	0.34 (0.34, 0.34)
10	0.35 (0.00, 0.65)	0.33 (0.33, 0.33)	**0.87 (0.86, 0.94)**	0.86 (0.86, 0.86)	0.36 (0.00, 0.65)	0.33 (0.33, 0.33)
11	0.35 (0.00, 0.65)	0.28 (0.28, 0.28)	0.88 (0.88, 0.94)	0.90 (0.90, 0.90)	0.36 (0.00, 0.65)	0.28 (0.28, 0.28)
12			0.89 (0.89, 0.90)	0.89 (0.89, 0.89)		
Low Tertile						
1	0.57 (0.52, 0.67)	0.59 (0.58, 0.60)	0.75 (0.64, 0.83)	0.74 (0.74, 0.75)	0.48 (0.44, 0.67)	0.57 (0.56, 0.57)
2	0.63 (0.55, 0.72)	0.63 (0.63, 0.63)	0.78 (0.68, 0.85)	0.78 (0.77, 0.78)	0.58 (0.53, 0.75)	0.63 (0.63, 0.64)
3	0.68 (0.60, 0.75)	0.66 (0.66, 0.67)	**0.80 (0.69, 0.96)**	0.78 (0.78, 0.78)	0.62 (0.54, 0.86)	0.69 (0.68, 0.69)
4	0.70 (0.62, 0.78)	0.69 (0.69, 0.69)	0.83 (0.70, 0.95)	**0.82 (0.82, 0.82)**	0.71 (0.56, 0.95)	0.74 (0.74, 0.74)
5	0.74 (0.70, 0.80)	0.72 (0.72, 0.72)	0.89 (0.70, 0.96)	0.84 (0.84, 0.84)	**0.91 (0.60, 0.94)**	0.79 (0.79, 0.79)
6	0.77 (0.71, 0.81)	0.74 (0.74, 0.74)	0.92 (0.70, 0.96)	0.86 (0.86, 0.86)	0.91 (0.68, 0.92)	**0.83 (0.83, 0.83)**
7	0.77 (0.73, 0.82)	0.76 (0.76, 0.76)	0.95 (0.86, 0.96)	0.88 (0.88, 0.88)	0.93 (0.72, 0.98)	0.86 (0.86, 0.86)
8	0.76 (0.75, 0.83)	0.78 (0.78, 0.78)	0.95 (0.87, 0.97)	0.90 (0.90, 0.90)	0.94 (0.89, 0.96)	0.89 (0.89, 0.89)
9	0.77 (0.77, 0.83)	**0.80 (0.80, 0.80)**	0.94 (0.94, 0.97)	0.91 (0.91, 0.91)	0.95 (0.89, 0.95)	0.91 (0.91, 0.91)
10	**0.84 (0.84, 0.91)**	0.85 (0.85, 0.85)	0.93 (0.93, 0.97)	0.92 (0.92, 0.92)	0.88 (0.88, 0.93)	0.88 (0.88, 0.88)
11	0.88 (0.85, 0.92)	0.88 (0.88, 0.88)	0.93 (0.93, 0.97)	0.95 (0.95, 0.95)	0.93 (0.87, 0.93)	0.90 (0.90, 0.90)
12	0.86 (0.86, 0.90)	0.87 (0.87, 0.87)	0.92 (0.92, 0.92)	0.92 (0.92,0.92)	0.86 (0.86, 0.94)	0.88 (0.88, 0.88)

Abbreviations: IQR Interquartile Range, CI Confidence Interval

Corresponding figure in main text, Figure 2. Bold values represent the point at which 0.80 is met or exceeded in each category

Table S3. Surrogate category analysis among males, Analysis 1

Repeat samples	Sensitivity		Specificity		Positive Predictive Value	
	Median (IQR)	Mean (95% CI)	Median (IQR)	Mean & CI	Median (IQR)	Mean & CI
High Tertile						
1	0.61 (0.56, 0.71)	0.66 (0.65, 0.67)	**0.81 (0.80, 0.87)**	**0.81 (0.82, 0.82)**	0.56 (0.53, 0.59)	0.57 (0.56, 0.58)
2	0.72 (0.71, 0.76)	0.74 (0.74, 0.74)	0.89 (0.88, 0.92)	0.88 (0.88, 0.89)	0.73 (0.68, 0.80)	0.72 (0.71, 0.73)
3	0.77 (0.75, 0.86)	0.78 (0.78, 0.79)	0.94 (0.91, 0.96)	0.92 (0.92, 0.92)	**0.86 (0.83, 0.88)**	**0.81 (0.80, 0.82)**
4	**0.80 (0.80, 0.90)**	**0.83 (0.82, 0.83)**	0.96 (0.96, 0.98)	0.95 (0.94, 0.95)	0.91 (0.91, 0.95)	0.86 (0.85, 0.87)
5	0.84 (0.80, 0.84)	0.85 (0.85, 0.86)	0.99 (0.98, 1.00)	0.97 (0.97, 0.97)	0.96 (0.96, 0.97)	0.90 (0.89, 0.90)
6	0.89 (0.84, 0.89)	0.89 (0.88, 0.89)	1.00 (1.00, 1.00)	0.99 (0.99, 0.99)	1.00 (1.00, 1.00)	0.95 (0.94, 0.96)
7	0.92 (0.91, 0.92)	0.93 (0.92, 0.93)	1.00 (1.00, 1.00)	1.00 (1.00, 1.00)	1.00 (1.00, 1.00)	0.99 (0.98, 0.99)
8	1.00 (0.93, 1.00)	0.97 (0.96, 0.98)	1.00 (1.00, 1.00)	1.00 (1.00, 1.00)	1.00 (1.00, 1.00)	1.00 (1.00, 1.00)
9-11	1.00 (1.00, 1.00)	1.00 (1.00, 1.00)	1.00 (1.00, 1.00)	1.00 (1.00, 1.00)	1.00 (1.00, 1.00)	1.00 (1.00, 1.00)
Medium Tertile						
1	0.33 (0.28, 0.42)	0.33 (0.32, 0.34)	**0.80 (0.74, 0.81)**	0.78 (0.77, 0.78)	0.53 (0.43, 0.57)	0.50 (0.48, 0.51)
2	0.59 (0.39, 0.62)	0.51 (0.51, 0.52)	0.78 (0.75, 0.85)	**0.80 (0.79, 0.80)**	0.69 (0.62, 0.71)	0.66 (0.65, 0.67)
3	0.51 (0.50, 0.72)	0.58 (0.57, 0.58)	0.83 (0.82, 0.86)	0.82 (0.82, 0.82)	0.75 (0.67, 0.76)	0.74 (0.73, 0.74)
4	0.57 (0.56, 0.69)	0.64 (0.64, 0.65)	0.87 (0.87, 0.88)	0.86 (0.86, 0.86)	**0.81 (0.73, 0.96)**	**0.83 (0.82, 0.83)**
5	0.62 (0.62, 0.91)	0.70 (0.70, 0.71)	0.90 (0.89, 0.90)	0.89 (0.89, 0.90)	0.85 (0.85, 1.00)	0.90 (0.89, 0.90)
6	0.71 (0.68, 0.95)	0.79 (0.79, 0.80)	0.94 (0.92, 0.94)	0.93 (0.93, 0.93)	1.00 (0.90, 1.00)	0.95 (0.94, 0.95)
7	**0.98 (0.73, 0.98)**	**0.88 (0.87, 0.89)**	0.97 (0.94, 0.97)	0.96 (0.96, 0.93)	1.00 (0.93, 1.00)	0.97 (0.97, 0.98)
8	0.99 (0.99, 0.99)	0.96 (0.95, 0.96)	0.99 (0.99, 0.99)	0.99 (0.99, 0.99)	1.00 (1.00, 1.00)	1.00 (1.00, 1.00)
9-11	1.00 (1.00, 1.00)	1.00 (1.00, 1.00)	1.00 (1.00, 1.00)	1.00 (1.00, 1.00)	1.00 (1.00, 1.00)	1.00 (1.00, 1.00)
Low Tertile						
1	0.75 (0.69, 0.82)	0.74 (0.73, 0.75)	0.75 (0.70, 0.81)	0.73 (0.73, 0.74)	0.62 (0.47, 0.71)	0.61 (0.60, 0.62)
2	**0.83 (0.79, 0.85)**	**0.80 (0.80, 0.81)**	**0.84 (0.72, 0.85)**	**0.80 (0.80, 0.80)**	0.70 (0.65, 0.80)	0.68 (0.67, 0.69)
3	0.85 (0.83, 0.96)	0.86 (0.86, 0.86)	0.80 (0.75, 0.90)	0.82 (0.92, 0.82)	0.73 (0.44, 0.76)	0.70 (0.69, 0.71)
4	0.90 (0.86, 1.00)	0.91 (0.91, 0.91)	0.83 (0.77, 0.87)	0.84 (0.93, 0.84)	0.76 (0.46, 1.00)	0.72 (0.71, 0.73)
5	0.95 (0.90, 1.00)	0.95 (0.94, 0.95)	0.84 (0.79, 0.91)	0.86 (0.85, 0.86)	**0.83 (0.48, 1.00)**	0.76 (0.75, 0.77)
6	0.94 (0.94, 1.00)	0.96 (0.96, 0.96)	0.86 (0.81, 0.95)	0.89 (0.89, 0.90)	1.00 (0.51, 1.00)	**0.83 (0.82, 0.84)**
7	0.97 (0.97, 0.97)	0.98 (0.98, 0.98)	0.98 (0.84, 0.98)	0.94 (0.93, 0.94)	1.00 (1.00, 1.00)	0.90 (0.89, 0.91)
8	0.99 (0.99, 0.99)	0.99 (0.99, 0.99)	0.99 (0.99, 0.99)	0.98 (0.97, 0.98)	1.00 (1.00, 1.00)	0.97 (0.96, 0.98)
9-11	1.00 (1.00, 1.00)	1.00 (1.00, 1.00)	1.00 (1.00, 1.00)	1.00 (1.00, 1.00)	1.00 (1.00, 1.00)	1.00 (1.00, 1.00)

Abbreviations: IQR Interquartile Range, CI Confidence Interval

Corresponding figure in main text, Figure 3

Bold values represent the point at which 0.80 is met or exceeded in each category

Table S4. Surrogate category analysis among males, Analysis 2

Repeat samples	Sensitivity Median (IQR)	Sensitivity Mean (95% CI)	Specificity Median (IQR)	Specificity Mean & CI	Positive Predictive Value Median (IQR)	Positive Predictive Value Mean & CI
High Tertile						
1	0.56 (0.42, 0.63)	0.52 (0.51, 0.53)	**0.80 (0.74, 0.81)**	0.77 (0.77, 0.78)	0.39 (0.33, 0.58)	0.44 (0.43, 0.45)
2	0.56 (0.50, 0.68)	0.59 (0.58, 0.59)	0.85 (0.78, 0.87)	**0.83 (0.83, 0.83)**	0.50 (0.42, 0.70)	0.56 (0.56, 0.57)
3	0.73 (0.63, 0.73)	0.67 (0.67, 0.67)	0.88 (0.81, 0.92)	0.86 (0.86, 0.87)	0.73 (0.62, 0.75)	0.67 (0.67, 0.68)
4	0.73 (0.48, 0.73)	0.66 (0.65, 0.66)	0.89 (0.86, 0.99)	0.88 (0.88, 0.89)	0.73 (0.48, 0.73)	0.66 (0.65, 0.67)
5	0.75 (0.75, 0.75)	0.75 (0.75, 0.75)	1.00 (1.00, 1.00)	0.96 (0.95, 0.96)	0.75 (0.75, 0.75)	0.75 (0.75, 0.75)
6			1.00 (1.00, 1.00)	1.00 (1.00, 1.00)		
Medium Tertile						
1	0.35 (0.12, 0.35)	0.28 (0.27, 0.29)	0.77 (0.71, 0.78)	0.75 (0.74, 0.75)	0.48 (0.13, 0.49)	0.38 (0.36, 0.39)
2	0.32 (0.24, 0.37)	0.31 (0.30, 0.32)	0.68 (0.68, 0.75)	0.71 (0.71, 0.72)	0.34 (0.27, 0.51)	0.35 (0.34, 0.37)
3	0.27 (0.10, 0.40)	0.30 (0.29, 0.31)	0.69 (0.64, 0.84)	0.73 (0.73, 0.74)	0.30 (0.01, 0.47)	0.33 (0.31, 0.34)
4	0.28 (0.00, 0.41)	0.30 (0.29, 0.31)	0.64 (0.64, 0.93)	0.76 (0.76, 0.77)	0.30 (0.40, 1.00)	0.33 (0.31, 0.34)
5	0.00 (0.00, 0.90)	0.28 (0.26, 0.30)	**0.95 (0.95, 0.97)**	**0.95 (0.94, 0.95)**	0.00 (0.00, 0.95)	0.29 (0.27, 0.31)
6			0.97 (0.97, 0.97)	0.97 (0.97, 0.97)		
Low Tertile						
1	0.57 (0.53, 0.71)	0.57 (0.56, 0.59)	0.70 (0.67, 0.72)	0.67 (0.66, 0.68)	0.55 (0.33, 0.62)	0.52 (0.50, 0.53)
2	0.61 (0.40, 0.73)	0.57 (0.56, 0.58)	0.70 (0.63, 0.74)	0.69 (0.69, 0.70)	0.60 (0.37, 0.73)	0.56 (0.54, 0.57)
3	0.61 (0.45, 0.90)	0.60 (0.59, 0.62)	0.76 (0.66, 0.84)	0.72 (0.72, 0.73)	0.61 (0.42, 0.84)	0.59 (0.58, 0.61)
4	0.46 (0.46, 0.98)	0.66 (0.64, 0.67)	0.68 (0.68, 0.87)	0.60 (0.59, 0.61)	0.44 (0.44, 1.00)	0.65 (0.64, 0.66)
5	**0.95 (0.95, 0.96)**	**0.96 (0.96, 0.96)**	0.00 (0.00, 0.90)	0.44 (0.42, 0.47)	**0.98 (0.92, 0.98)**	**0.96 (0.96, 0.97)**
6	0.97 (0.97, 0.97)	0.97 (0.97, 0.97)			0.97 (0.97, 0.97)	0.97 (0.97, 0.97)

Abbreviations: IQR Interquartile Range, CI Confidence Interval

Corresponding figure in main text, Figure 4

Bold values represent the point at which 0.80 is met or exceeded in each category
